# Explainable hybrid vision transformers and convolutional network for multimodal glioma segmentation in brain MRI

**DOI:** 10.1038/s41598-024-54186-7

**Published:** 2024-02-14

**Authors:** Ramy A. Zeineldin, Mohamed E. Karar, Ziad Elshaer, Jan Coburger, Christian R. Wirtz, Oliver Burgert, Franziska Mathis-Ullrich

**Affiliations:** 1 Department Artificial Intelligence in Biomedical Engineering (AIBE), Friedrich-Alexander-University Erlangen-Nürnberg (FAU), 91052 Erlangen, Germany; 2 Research Group Computer Assisted Medicine (CaMed), Reutlingen University, 72762 Reutlingen, Germany; 3 Faculty of Electronic Engineering (FEE), Menoufia University, Minuf, 32952 Egypt; 4 Department of Neurosurgery, University of Ulm, 89312 Günzburg, Germany

**Keywords:** Biomedical engineering, Cancer, Medical imaging

## Abstract

Accurate localization of gliomas, the most common malignant primary brain cancer, and its different sub-region from multimodal magnetic resonance imaging (MRI) volumes are highly important for interventional procedures. Recently, deep learning models have been applied widely to assist automatic lesion segmentation tasks for neurosurgical interventions. However, these models are often complex and represented as “black box” models which limit their applicability in clinical practice. This article introduces new hybrid vision Transformers and convolutional neural networks for accurate and robust glioma segmentation in Brain MRI scans. Our proposed method, TransXAI, provides surgeon-understandable heatmaps to make the neural networks transparent. TransXAI employs a post-hoc explanation technique that provides visual interpretation after the brain tumor localization is made without any network architecture modifications or accuracy tradeoffs. Our experimental findings showed that TransXAI achieves competitive performance in extracting both local and global contexts in addition to generating explainable saliency maps to help understand the prediction of the deep network. Further, visualization maps are obtained to realize the flow of information in the internal layers of the encoder-decoder network and understand the contribution of MRI modalities in the final prediction. The explainability process could provide medical professionals with additional information about the tumor segmentation results and therefore aid in understanding how the deep learning model is capable of processing MRI data successfully. Thus, it enables the physicians’ trust in such deep learning systems towards applying them clinically. To facilitate TransXAI model development and results reproducibility, we will share the source code and the pre-trained models after acceptance at https://github.com/razeineldin/TransXAI.

## Introduction

Intra-axial brain tumors are among the ten most common malignancies leading to death^1^. Although there are no screening or preventive examinations, effective diagnosis, and therapy influence the further course of gliomas. Neurosurgical intervention is the first and sometimes the only therapy for many types of gliomas^2^. In particular, the precise localization of pathological structures (lesions) within the brain anatomy is a major issue in neurosurgery. This challenge is related to the difficulty in visually delineating these pathological targets from the healthy brain parenchyma.

Magnetic resonance imaging (MRI) is the preferred modality for the evaluation of intra-axial, identification of normal brain structures, peritumoral edema, and detection of tumor-infiltrated regions^3^. In particular, multimodal MRI of the brain, including native T1-weighted (T1), post-contrast (T1Gd (Gadolinium)), T2-weighted (T2), and T2-weighted fluid-attenuated inversion recovery (FLAIR) sequences, is the gold standard to detect brain gliomas including their sub-regions^4^. The presence of peripheral contrast enhancement, central necrotic areas, intra-tumoral hemorrhages, ill-defined infiltration, and extensive perifocal edema is commonly seen in aggressive lesions which raises the possibility of high-grade glioma (HGG) or glioblastoma (GBM) (WHO grade IV). However, non-enhancing tumor regions raise the possibility of low-grade gliomas (LGG). The Multimodal Brain Tumor Segmentation Challenge (BraTS) 2019 dataset^5–7^ provides a multi-institutional annotated MRI dataset aiming at performance evaluation of state-of-the-art (SOTA) automated deterministic solutions for the segmentation of intra-axial brain lesions.

Recent developments in deep learning, specifically convolutional neural networks (CNN) have achieved excellent performance for processing and analyzing medical images, including those associated with brain tumor segmentation^8,9^, image registration^10,11^, and image classification^12^. In particular, the convolutional encoder-decoder architectures, U-Net^13,14^, have revolutionized the medical field with outstanding feature representation capabilities. In a typical U-shaped architecture, the encoder is a series of convolutional layers each followed by down-sampling layers for feature representation learning with local receptive fields. The decoder aims at up-sampling the extracted deep feature maps to the same size as the original input. By using skip connections, the U-Net fuses the feature representations at different resolutions of the encoder with the corresponding layers at the decoder to recover spatial information that is lost during down-sampling.

Following this U-Net architecture, Zhou et al. proposed UNet++^15^ by redesigning skip connections to aggregate multiple-scale feature maps of an ensemble of U-Nets co-learning using deep supervision. Res-UNet^16^ was proposed to address small thin structures using a weighted attention mechanism and ResNet-based^17^ skip connection scheme. Similarly, KiU-Net^18^ employs an overcomplete convolutional architecture to effectively identify smaller structures and achieve precise segmentation of boundary regions by restricting the expansion of the receptive field size. It is worth mentioning that all these methods are based on CNNs, i. e. rely on the convolutional operation to capture local features by gathering information from neighborhood pixels. So, they lack the ability to capture long-range dependency explicitly although there are some recent works trying to model global context for CNN such as^19,20^ without providing satisfying results in modeling long dependencies.

Lately, Transformer has achieved tremendous success in the natural language processing (NLP) field^21^. The self-attention mechanism in Transformer allows to model correlations among all the input tokens and hence is superior to CNN in handling long-range dependencies. Vision Transformer (ViT)^22^ is a good example, which achieved SOTA on ImageNet classification. By reshaping input images into 2D patches with positional embeddings, ViT achieved comparable performance with the CNN-based methods. Some approaches utilized Transformers as feature extractors or in the middle bottleneck layers^23,24^. TransUNet^23^ is the first attempt to combine Transformer with U-Net to establish self-attention for medical image segmentation. TransUNet uses a CNN encoder which generates feature maps to be fed into the Transformer using patch embedding in the bottleneck. TransAttUNet^24^ integrated multi-level guided attention U-Net with Transformer to enhance the performance of medical image segmentation. Though achieving satisfying results, these methods heavily rely on a self-attention mechanism which would suffer from tremendous computation requirements at high-resolution volumes such as MRI images.

In addition, pure transformer-based architectures were proposed, such as SwinUNet^25^ utilizes Swin Transformer as the building block for a U-shaped pure Transformer Encoder-Decoder architecture based on the shifted windows mechanism. The primary constraint for the use of pure Transformers is the need for huge training datasets (14M-300M images) which is not always available, especially in the medical field. This is because Transformers lack inductive biases, e. g. localized receptive fields, in contrast to the CNN models, and therefore do not generalize well to test cases when trained on smaller data.

Nonetheless, most machine learning and/or deep learning techniques are under development for deployment in the clinical field^26,27^. The primary reason behind that is the “black box” nature of the deep models which are often characterized by the lack of human-like explainable decisions. In addition, these models include a substantial number (within millions) of extracted feature maps in each internal layer which are assumed to contain meaningful information about the input problem and its possible solution. This makes fully understanding DL methods highly problematic, even for professional experts. Thus, the application of such “black box” models in highly sensitive medical applications is very limited^26,28^. Recently, there is a growing interest in explainable AI (XAI) to address the justification of the decision-making process made by DL models^29^. Though the explainability provides no improvement in the accuracy of the deep learning model, XAI is important to guarantee safety during clinical application and increase the trust of clinical end users, i.e., surgeons and radiologists. XAI provides machine learning methods the ability to describe their “black box” nature in explainable or interpretable terms to humans^26,28^.

In previous studies, several interpretability methodologies have been introduced to explain the behavior of machine learning methods in medical applications, such as COVID-19 diagnosis^30^, retinal imaging^31^, and skin cancer^32^. Also, some research works have been conducted to generate explainable results for brain tumor segmentation networks. In^33^, Pereira et al. employed a joint Restricted Boltzmann Machine system (RBM) and a Random Forest (RF) classifier to enhance the interpretability of a machine learning system. Inspired by^34^, they provided two levels of interpretation, i.e. local and global, allowing for an evaluation of the extracted task-specific features and the voxel-level predictions, respectively. A key limitation of their mutual RBM-RF feature selection strategy is the randomness of the input feature vector in each node which can be computationally expensive for medical imaging tasks and, therefore, time-consuming.

In^35^, a method has been developed for visual explanations towards explaining the “black box” nature of CNNs. This method extended Class Activation Mapping (CAM)^36^ to extract explanations to interpret a segmentation network for brain tumors in MRI. Moreover, they investigated how the input MRI modality perturbation affects the prediction strategy of different brain lesion sub-regions. However, standard CAM approaches are restricted to a certain type of CNNs without including any multimodal input or fully connected layers CNNs.

Li et al. developed an explainable ensemble Gaussian kernel (XEGK) to substitute for CNN in feature extraction, in which they used a Gaussian kernel to capture characteristic features from relevant regions of the input^19^. They applied their method to mono-channel input and multi-channel inputs by leveraging the Gaussian mixture model (GMM) and fusion of multiple GMMs, respectively. To interpret the experimental results, they used Shapely additive explanations (SHAP)^37^ to reflect the features’ contribution. SHAP is a perturbation-based approach from the coalitional game theory which assigns a feature importance value for each class prediction. It is therefore inefficient in critical medical applications since the network must be run for the number of samples multiplied by the number of features.

Natekar et al. generated visual explanations of three deep neural networks (DNN) for the segmentation of brain tumors^38^. They applied Grad-CAM^36^ to explain the contribution of the internal layers of those segmentation networks helping to understand “why” DNN achieved quantitively highly accurate tumor segmentations. The experiments indicated that DNN follows a human-like hierarchical approach for localizing different parts of the brain tumor.

Overall, the main focus of recent XAI research in medical image segmentation has been on integrating visual interpretability without considering the clinical evaluation of the resultant visualizations. Besides, less attention has been paid to the inclusion of medical knowledge into the decision approach made by AI-based models. Moreover, the decisions of these models must be consistent with the clinical knowledge to gain the trust of medical professionals and encourage them to adopt AI-based systems.

In this work, TransXAI framework is proposed to leverage the power of CNN and Transformers as a hybrid model for explainable glioma segmentation. In designing our hybrid CNN-Transformer model, we carefully considered the specific challenges of glioma segmentation, opting for a fusion of architectures that harnesses the strengths of both local and global feature extraction to provide a comprehensive understanding of MRI scans. In particular, CNN is employed as an encoder to extract local image representations while a ViT is utilized to further the long-range dependency. The contributions of this study are divided into four-fold:

- A hybrid CNN-Transformer architecture is proposed for the segmentation of brain tumors, which combines high-resolution local representations from CNN and the long-range dependency captured by Transformers.
- An effective XAI diagnosis generator has been developed to extract explanations from the medical segmentation network.
- Evaluation of the proposed TransXAI framework on the multimodal brain tumor segmentation dataset demonstrates its effectiveness, superiority, and robustness.
- Explainability-driven evaluation by clinical experts showed that the proposed approach increases surgeons’ trust in deep learning systems by providing evidence linked to the results of our TransXAI from the surgical point of view.

## Results

### Segmentation results

**Figure 1:**
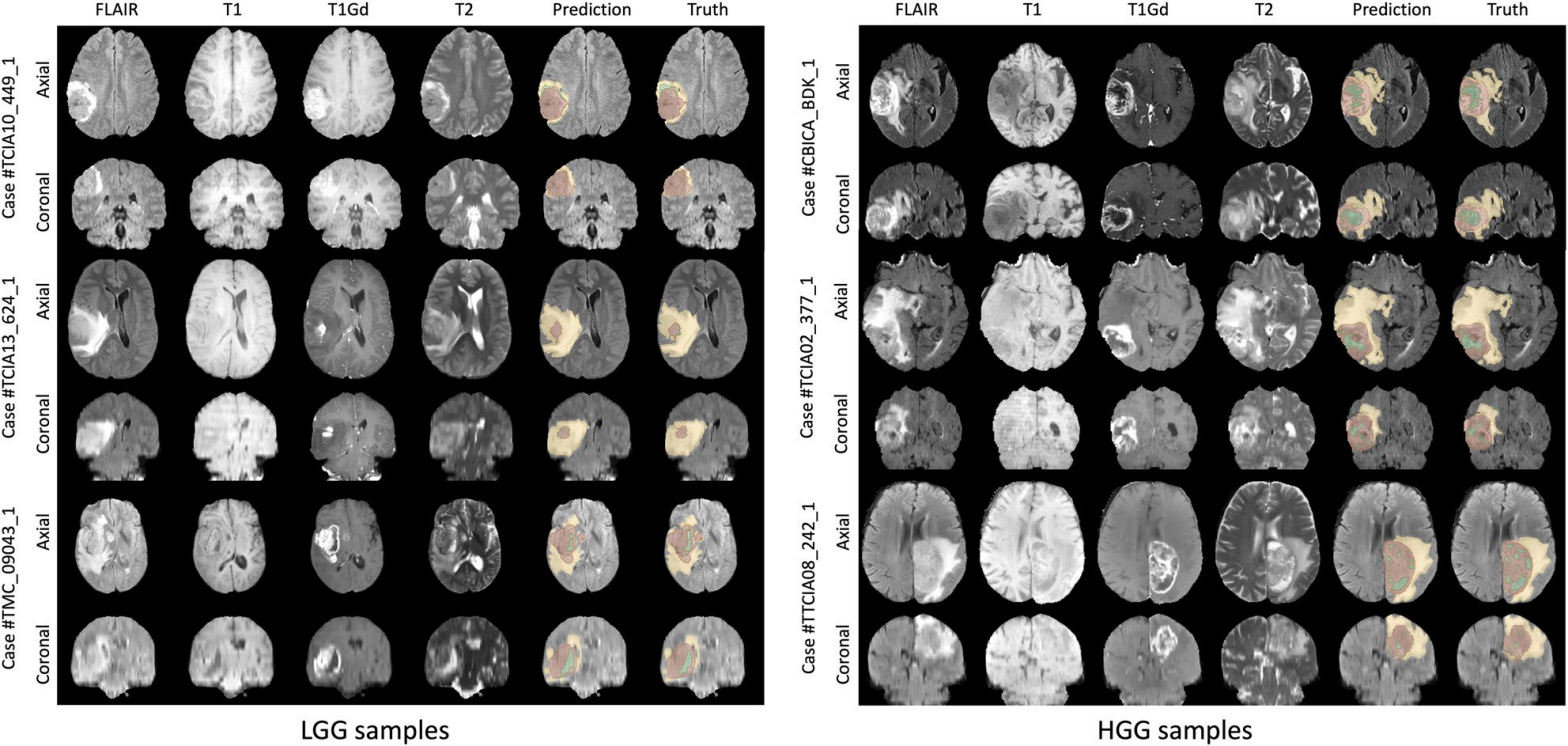
Visual segmentation results of TransXAI on Axial and Coronal views along with the results of predictions for three BraTS 2019 Challenge LGG samples (*left*) and HGG samples (*right*). The tumor regions are color-coded, with the ET shown in *green*, the TC including both *green* and *red* regions, and the WT representing all the segmentation classes.

Figure 1 shows the visual segmentation results of the proposed TransXAI for three HGG and three LGG of the BraTS 2019 training set. In this Figure, the input MRI slices and the predicted segmentation maps overlaid on the FLAIR MRI are presented in the Axial and Coronal views. The results demonstrate that our proposed model shows competitive performance, especially in detecting the brain glioma boundaries and its sub-regions. Further, the statistical results reported by the BraTS evaluation platform^5^ confirm this finding as Table 1 lists the average dice similarity coefficient (DSC) and Hausdorff distance (95%) (HD95) for our TransXAI model on the BraTS 2019 validation set.

**Table 1:** The fivefold cross-validation STAPLE ensemble results of the TransXAI on the BraTS 2019 validation, along with the results of single folds.

Model	DSC			HD95		
	ET	TC	WT	ET	TC	WT
Fold 0	0.725	0.767	**0.883**	6.81	9.66	**6.35**
Fold 1	0.730	0.770	0.869	9.11	11.37	7.68
Fold 2	0.720	0.777	0.876	6.08	8.23	5.52
Fold 3	**0.746**	0.758	0.868	4.93	8.76	6.73
Fold 4	0.734	0.756	0.874	5.05	8.67	6.62
Ensemble	0.745	**0.782**	0.882	**4.31**	**7.90**	6.36

Bold represents the best value within each metric column. The metrics include Dice Similarity Coefficient (DSC), and Hausdorff Distance at 95% (HD95) across all sub-regions.

Furthermore, we have undertaken additional experiments to evaluate the robustness of our TransXAI model. These experiments involved a meticulous assessment of the model's performance across the five folds of cross-validation, which provides a comprehensive view of its consistency and resilience against variations in the training data. Table 1 showcases the 5-fold cross-validation ensemble results of the TransXAI method on the BraTS 2019 validation dataset, along with the results of individual folds. (After the second BraTS external validation) The consistent performance of TransXAI across different folds of cross-validation underscores its robustness, an essential attribute for clinical applications where variability in data is commonplace

The TransXAI method achieved a DSC of 0.745 for the enhancing tumor (ET) region, 0.782 for the tumor core (TC) region, and 0.882 for the whole tumor (WT) region, with an average DSC of 0.803. Additionally, it achieved an HD95 of 4.31 mm for ET, 7.90 mm for TC, 6.36 mm for WT, and an average HD95 of 6.192 mm. These results provide a comprehensive view of the method's performance across different folds, emphasizing its consistency and robustness in glioma sub-region segmentation.

Our methodological choices, including the application of specific data augmentation techniques and the selection of a 5-fold cross-validation approach, were driven by the need to build a model that is not only accurate but also generalizable across different data distributions. Comparing these results with the SOTA methods, TransXAI method demonstrated competetive performance in various aspects. While it excels in certain metrics, such as TC DSC and HD95 across all three subregions, it closely aligns with other leading methods in terms of ET and WT DSC, as indicated in Table 2. The average DSC of 0.803 and average HD95 of 6.19, while notable, reflect a competitive standing rather than a clear superiority.

**Table 2:** Comparison of the segmentation results of TransXAI and SOTA on the BraTS 2019 validation set.

Method	DSC				HD95			
	ET	TC	WT	Avg	ET	TC	WT	Avg
U-Net^9^			0.813				19.75	
3D U-Net^14^	0.709	0.725	0.874	0.769	*5.062*	8.719	9.43	7.74
KiU-Net^18^	0.664	0.706	0.861	0.744	9.418	13.04	12.79	11.75
Res U-Net^16^	0.667	0.706	0.853	0.742	7.270	9.57	8.55	8.46
MS U-Net^39^	0.713	0.711	0.865	0.763	8.2465	12.65	9.42	10.11
Attention U-Net^40^	**0.7596**	0.772	**0.888**	**0.807**	5.202	*8.26*	7.76	7.07
mmFormer^41^	0.60	0.73	0.829	0.720				
V-Net^42^	0.739	0.766	*0.887*	0.797	6.131	8.71	**6.26**	*7.03*
Starke et al.^43^	0.710	0.710	0.850	0.757	6.57	10.28	8.85	8.57
TransXAI (Ours)	*0.745*	**0.782**	0.882	*0.803*	**4.31**	**7.90**	*6.36*	**6.19**

Bold and italic represent the best and second-best within each metric column, respectively.

### External multi-site validation

To validate the generalizability of our TransXAI method, we have extended our evaluation to include external datasets from the FeTS2022 Challenge, which is based on the BraTS2021 Challenge dataset^7,44,45^. This dataset is a significant compendium of 1251 multi-modal brain MRI scans, inclusive of T1, T1ce (post-contrast T1-weighted), T2, and FLAIR sequences, each of a uniform size of 240 × 240 × 155 and an isotropic resolution of 1mm3 per voxel. Accompanying these images are multi-label tumor segmentation masks, distinguished into four distinct categories: background, ET, TC, and WT. The dataset mirrors real-world diversity from different multi-site institutions, thus providing a heterogeneous mix of imaging protocols and patient demographics. This feature enables the assessment of TransXAI in a simulated multi-site learning environment, which is a step closer to its deployment in clinical practice.

In conducting our external validation, we have been meticulous in selecting data from the FeTS2022 Challenge that minimizes overlap with our training set from BraTS 2019. Detailed attention was given to the case distribution across institutions to ensure the independence of the validation datasets. Specifically, we have avoided using cases from institutions that contributed to the BraTS 2019 dataset except for a controlled number from Institute 1, which is sufficiently justified by the majority of new cases ensuring a valid assessment (with only 129 overlapping cases out of 511). All other institutes (2, 18, 20, 21, 22) have no cases included in BraTS 2019, ensuring the integrity of the validation process.

Following the BraTS testing procedure, we have performed rigorous evaluations to assess the precision of our TransXAI model in delineating the ET, TC, and WT regions. The results of this evaluation are critical, as they directly inform the potential clinical utility of our model. The results are summarized in Table 3. By utilizing the commonly utilized metrics, DSC and HD95, we were able to capture a comprehensive picture of TransXAI's segmentation performance. In particular, the results indicate high accuracy and robustness in identifying tumor boundaries and consistency across different tumor subregions. This external validation against FeTS2022 datasets underscores the potential of TransXAI for clinical integration. The detailed analysis of our model's performance has identified key areas for future enhancement, particularly in addressing the variability across different imaging protocols and patient demographics. These aspects are critical for developing more adaptable and robust AI systems for real-world clinical use.

**Table 3:** External validation of TransXAI on FeTS2022 challenge datasets.

Institute	Number of cases	BraTS 2019 Overlap	DSC				HD95			
			ET	TC	WT	Avg	ET	TC	WT	Avg
1	511	129	0.710	0.891	0.739	0.780	4.68	14.95	7.88	9.17
2	6	–	0.638	0.913	0.646	0.732	8.01	10.69	14.14	10.95
18	382	–	0.687	0.854	0.718	0.753	6.20	16.77	9.04	10.67
20	33	–	0.759	0.946	0.784	0.830	4.69	7.15	7.43	6.42
21	35	–	0.804	0.931	0.800	0.845	6.46	9.12	8.68	8.09
22	7	–	0.778	0.897	0.823	0.833	5.29	13.66	7.30	8.75

DSC and HD95 are reported for enhancing tumor (ET), tumor core (TC), and whole tumor (WT), along with their average (Avg) values for each institute.

### Role of MRI in tumor detection

**Figure 2:**
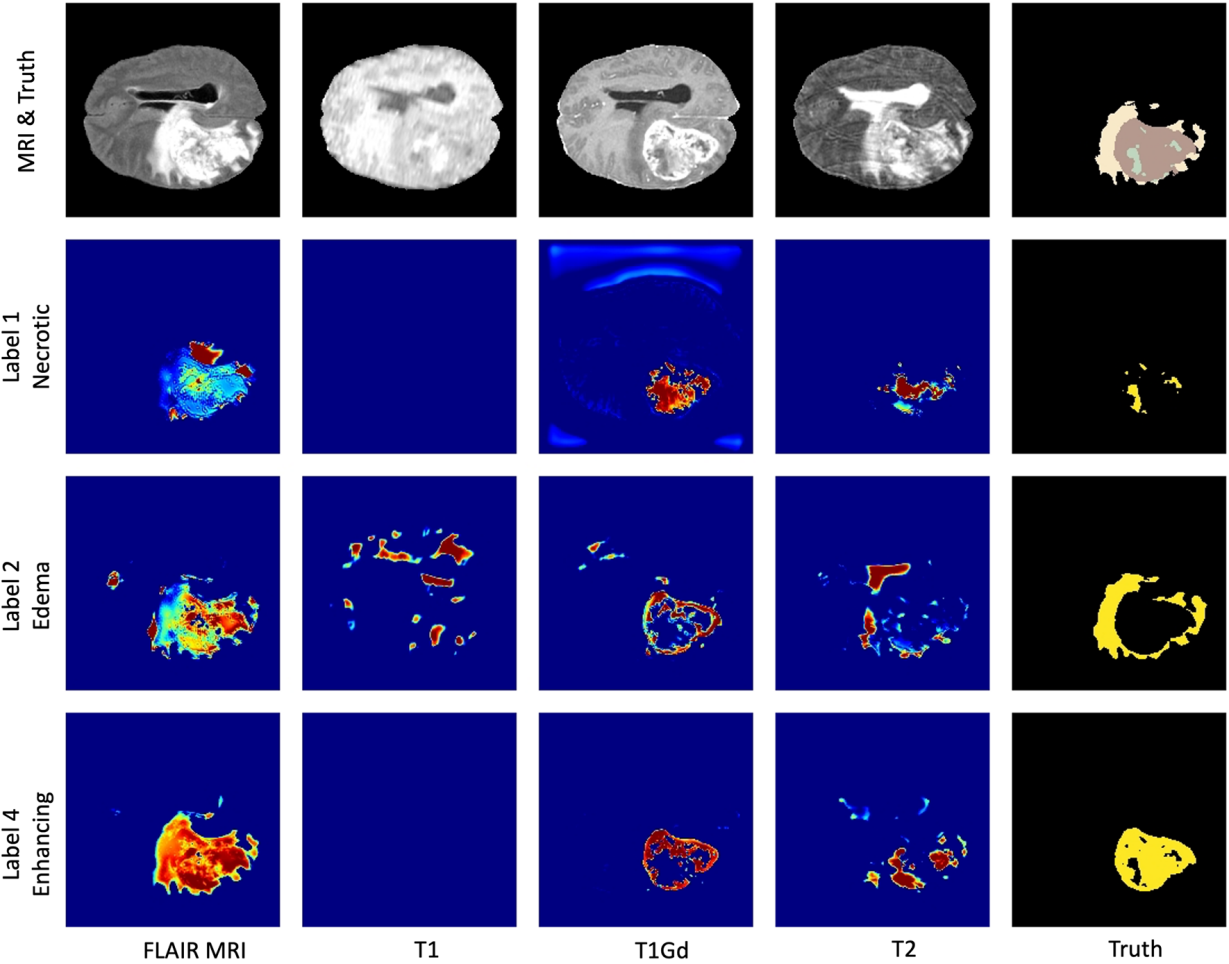
Impact of MRI input modality in the detection of different tumor labels. The first row shows the input MRI sequences and the ground truth annotations. The following rows correspond to label 1 (the necrotic tumor core), label 2 (the peritumoral edema), and label 4 (the enhancing tumor). In the saliency maps, warmer regions represent a high score for the specified label detection.

To better interpret the behavior of the CNN model, we performed a further experiment for generating visual explanations of every tumor class using Grad-CAM. We experimented to infer TransXAI with a specified MRI modality without involving other MRI sequences. This led to understanding the importance of each MRI input, namely, T1, T1Gd, T2, and FLAIR in the process of different tumor label localization. Figure 2 outlines the visual representation captured by the output convolutional layer of our TransXAI model with respect to the input MRI modality. The results demonstrate that the detection of each tumor sub-region is related to one or more of the input MRI volumes coherent with expert radiologists’ and raters’ observations in reference^6^. For instance, T1Gd and T2 contribute most to the detection of the gross TC, including both label 1 (NC) and label 4 (ET), while the edema and the WT region are predicted using FLAIR. Though, the visual explanations of T1 are the least important maps with very little contribution to the tumor sub-components segmentation and could, therefore, be removed for computational performance advantage without model accuracy degradation.

### Grad-CAM for different CNN layers

**Figure 3:**
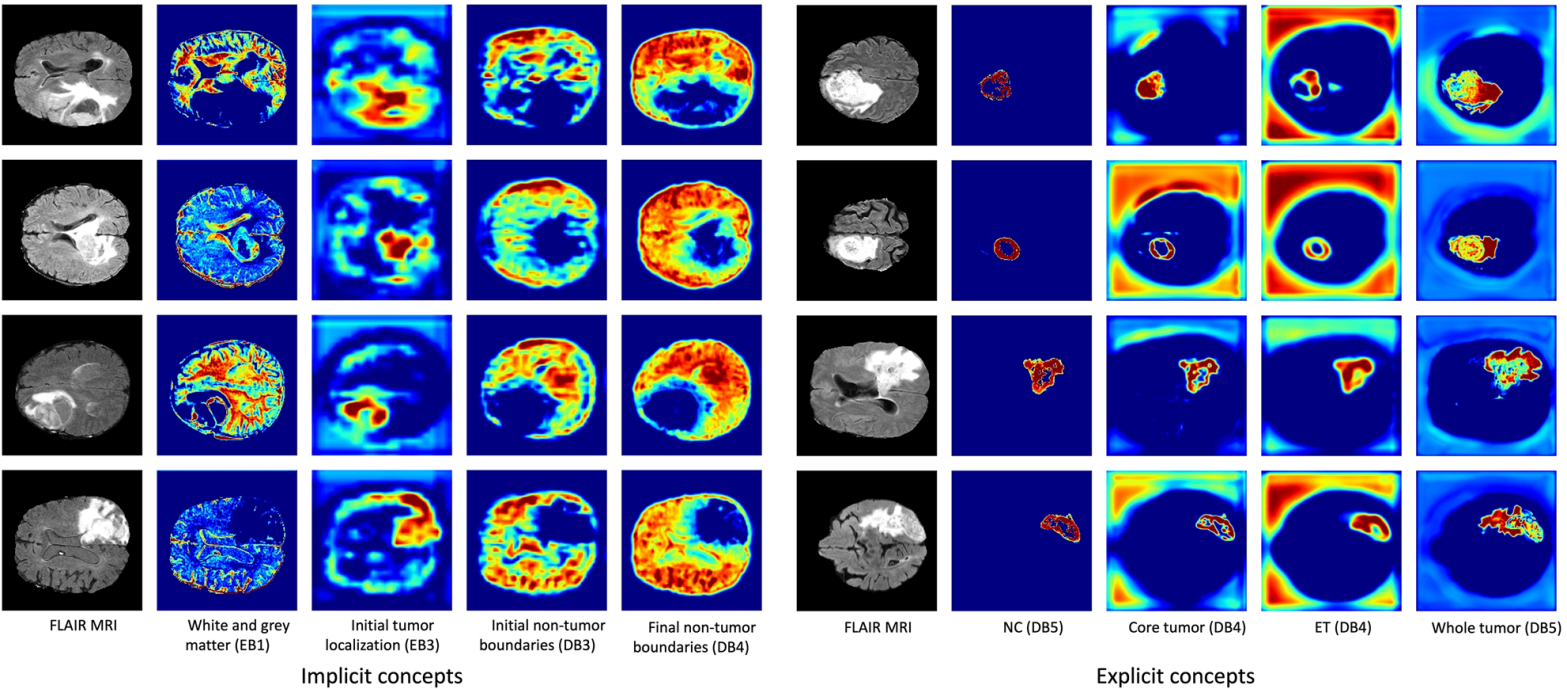
Saliency maps for implicit concepts (*left*) and explicit concepts (*right*) learned by individual filters of the CNN model. It is interesting to note that there are no labels for implicit concepts in the training dataset. Warmer regions represent a high score for the specified concept in the prediction map. Note that EB and DB denote the encoder and decoder block layers, individually.

In this section, Grad-CAM has been applied to interpret the proposed TransXAI for tumor segmentation. Figure 3 shows saliency maps for the internal convolutional layers of the investigated CNN model. These visual explanations provide details on the information flow inside individual filters of the network and how it learns some meaningful concepts. In this hybrid network, the encoder typically consists of successive layers to capture contextual information, Transformer blocks embedded in the bottleneck, and the expanding decoder path contains upsampling operators to enable high-resolution localization of the target tumor voxels.

It is important to observe that, internal layers of the deep neural network learn some implicit as well as explicit concepts although the training stage included only explicit tumor labels. For example, Figure 3 (a) demonstrates how the model implicitly differentiates white matter and gray matter region in encoder block 1 which the network has not been trained to learn. Similarly, the network understands other implicit concepts such as initial and final non-tumor boundaries in decoder block 3 and block 4, correspondingly. In addition, the CNN model learns explicit brain tumor sub-regions which are labeled in the training dataset as depicted in Figure 3 (b).

Furthermore, experimental results show that our proposed network follows a top-down approach for detecting and segmenting brain glioma. First, the model starts with learning the entire brain tissue, followed by the initial tumor boundaries, and finally, small objects and fine details are localized. In Figure 3 (b), some examples of finer segmentations are presented for the expansive path. Such filters outline the NC (label 1) in decoder block 5, the ET (label 4) in decoder block 4, the TC region (label 1 and label 4) in decoder block 4, and the WT region (all labels) in decoder block 5. Our findings are consistent with the top-down coupling approach that the human brain follows for the comprehension of relevant visual features in global first then local areas^46^.

### Clinical feedback

In the medical domain, explainable and interpretable AI systems are crucial for many applications such as research, education, and clinical treatments^47^. XAI systems can support medical professionals to understand the prediction process followed by deep learning models, and thus, enhance human experts’ trust in the system’s decisions. XAI systems can significantly enhance the capabilities of medical professionals in understanding the prediction process followed by deep learning models, thus fostering increased trust in the system's decisions.

To quantitatively assess the clinical utility of our TransXAI model, structured interviews were conducted with two medical experts from the Department of Neurosurgery at Ulm University Hospital, each possessing extensive clinical experience—ten and seven years, respectively—to critically evaluate the practical utility of TransXAI. Experts were asked to assess the algorithm based on several criteria, including the accuracy of segmentation against known clinical cases, the clarity and clinical relevance of heatmaps, and the potential for the method to enhance current diagnostic and treatment planning procedures. Their evaluation involved a review of output from the TransXAI model in comparison with actual patient cases, discussions on the interpretability of the model’s decision-making process, and the alignment with their clinical experience.

Our clinical collaborations revealed several potential benefits of our proposed hybrid CNN-Transformer architecture for real-world clinical applications. One of the key advantages lies in the enhanced interpretability of the segmentation results through the application of the Grad-CAM technique. Medical experts found Grad-CAM to be a valuable tool for understanding the model's decision-making process. This interpretability not only enhances transparency in "black box" machine learning systems but also provides human-understandable insights into the reasoning behind the model's predictions.

Furthermore, our method's capability to generate spatial attention maps and implicit concept saliency maps aligns well with the diagnostic practices of medical specialists. The logical systematic process exhibited by TransXAI during the segmentation process mimics the cognitive approach used by clinicians to identify various tissue structures, such as neoplastic tissues and perifocal edema. This congruence between model behavior and clinical practice fosters a familiarity for medical specialists, facilitating more effective interaction with AI-assisted segmentation results.

However, it is essential to acknowledge certain limitations and considerations when applying our architecture in clinical scenarios. The performance of AI models can be influenced by factors such as dataset variability, acquisition protocols, and specific clinical contexts. While our approach achieved competitive segmentation results, potential inconsistencies in segmentation accuracy may arise due to the diversity of tumor characteristics and imaging conditions across different patient cohorts. Additionally, the reliance on specific MRI modalities for accurate segmentation raises the need for careful selection of imaging protocols to optimize the clinical utility of our method.

In conclusion, our hybrid CNN-Transformer architecture, in conjunction with the interpretability afforded by Grad-CAM and implicit concept saliency maps, holds promise for enhancing clinical decision-making and research efforts in glioma segmentation. The collaboration between AI systems and medical experts is key to maximizing the benefits of such technologies while navigating their limitations. Further investigations and validations within diverse clinical settings are essential to comprehensively assess the performance and robustness of our approach in real-world applications.

## Discussion

Our study has yielded comprehensive insights into the performance and interpretability of the proposed TransXAI architecture for glioma sub-region segmentation in multimodal brain MRI scans. We have further clarified the TransXAI model's decision-making process, which is twofold: first, it employs CNNs for precise local feature detection; then, it uses Transformers to contextualize these features globally, mirroring the holistic approach a surgeon often takes when assessing MRI scans.

Through the segmentation results presented in Figure 1, we have demonstrated the outstanding performance of TransXAI in detecting brain glioma boundaries and their sub-regions. This is supported by statistical metrics in Table 1, which provides detailed insights into our TransXAI model's performance metrics across the different cross-validation folds on the BraTS 2019 validation set. Additionally, a comparative analysis in Table 2 highlights the competitive performance of TransXAI against SOTA methods, indicating its efficacy in accurately capturing the intricate details of glioma sub-regions.

The robustness of the TransXAI model has been rigorously evaluated through five-fold cross-validation experiments, demonstrating its consistent performance across different folds. The ensemble model's results in Table 1 underscore the reliability of the method in glioma sub-region segmentation, with promising DSC and HD95 values across ET, TC, and WT regions. These results emphasize the robustness of the method and its capability to generalize across varying data.

Another notable aspect of the proposed approach is its ability to interpret the behavior of the CNN model using an XAI technique, namely the Grad-CAM. By inferring TransXAI with specific MRI modalities, we have discerned the importance of individual MRI inputs for different tumor label localizations. This analysis, outlined in Figure 2, provides insights into the distinct contributions of each MRI sequence, which align with the observations of expert radiologists. Our findings emphasize the significance of utilizing FLAIR and T2 MRI scans for precise estimation of perifocal edema and the importance of T1Gd for delineating high-grade intra-axial lesions. The implications for low-grade lesions are also highlighted, providing valuable guidance for selecting the appropriate MRI sequences in clinical scenarios. This information holds significant importance for model developers, enabling them to assess the impact of selectively including or excluding specific sequences during the training process and analyzing their effect on segmentation accuracy, and medical practitioners. It facilitates a deeper understanding of the internal decision-making process of the DL model and its intricate interactions with distinct imaging modalities.

To understand the model internal information workflow, we have investigated the interpretation of individual filters through saliency maps. As depicted in Figure 3, our analysis shows that the model learns implicit and explicit concepts. The presence of implicit concepts, such as differentiating white and gray matter, indicates the model's capacity to discern meaningful features beyond its explicit training labels. This exploration of the CNN's systematic segmentation process has shown alignment with the clinical practice of identifying neoplastic tissues and differentiating between brain structures. The provision of activation maps from internal filters enhances transparency and confidence in the model's predictions. This is particularly valuable in generating human-understandable interpretations that can assist medical specialists in evaluating segmentations for clinical trials.

To ensure a robust clinical validation, the engagement with medical experts was structured around specific clinical use cases, with experts providing their assessment on a case-by-case basis. This included detailed discussions on the segmentation results for a range of glioma sub-types and complexities. The clinical implications of our work are evident in the feedback provided by medical professionals and the consensus reached on the utility of TransXAI in a clinical setting. Our approach to XAI aligns with the requirements of the medical domain, where interpretability and transparency are critical. Grad-CAM explanations have been well-received, as they offer intuitive visualizations that are easily understood from a surgical standpoint. The decision-making process within our TransXAI model is grounded in both data-driven insights and clinical interpretability. We provide an in-depth analysis of how the model interprets features aligning with clinical expectations and diagnostic criteria for gliomas. This paves the way for improved collaboration between AI models and medical experts, fostering trust and facilitating the integration of AI-based tools into clinical workflows.

Within the scope of our current investigation, our focus has centered on glioma segmentation utilizing 2D axial MRI slices. Notably, the selection of axial slices represents intrinsic clinical significance and methodological considerations. The choice of axial slices is well-grounded in both clinical relevance and practical considerations. Axial images are widely used in clinical practice for brain imaging due to their compatibility with anatomical landmarks and consistent visualization of structures. Additionally, the spatial distribution of brain gliomas often follows the axial plane. This alignment is particularly relevant for accurate tumor boundary delineation and understanding the extent of tumor involvement in adjacent regions. Furthermore, axial slices are commonly available in medical datasets, including the BraTS dataset, simplifying data collection and preprocessing.

Nevertheless, a considerable interest lies in extending our methodology to accommodate 3D volumetric inputs. The transition to 3D data introduces a compelling dimension of exploration, with the potential to harness inherent spatial context and elevate the accuracy of glioma delineation. However, it is essential to acknowledge that this transition comes accompanied by its constellation of challenges, including heightened computational demands and the intricate integration of spatial information across multi-dimensional slices. This consideration steered our choice toward 2D slices, aligning with the pragmatic necessity to strike a symmetry between computational performance and model complexity.

## Materials and methods

**Figure 4:**
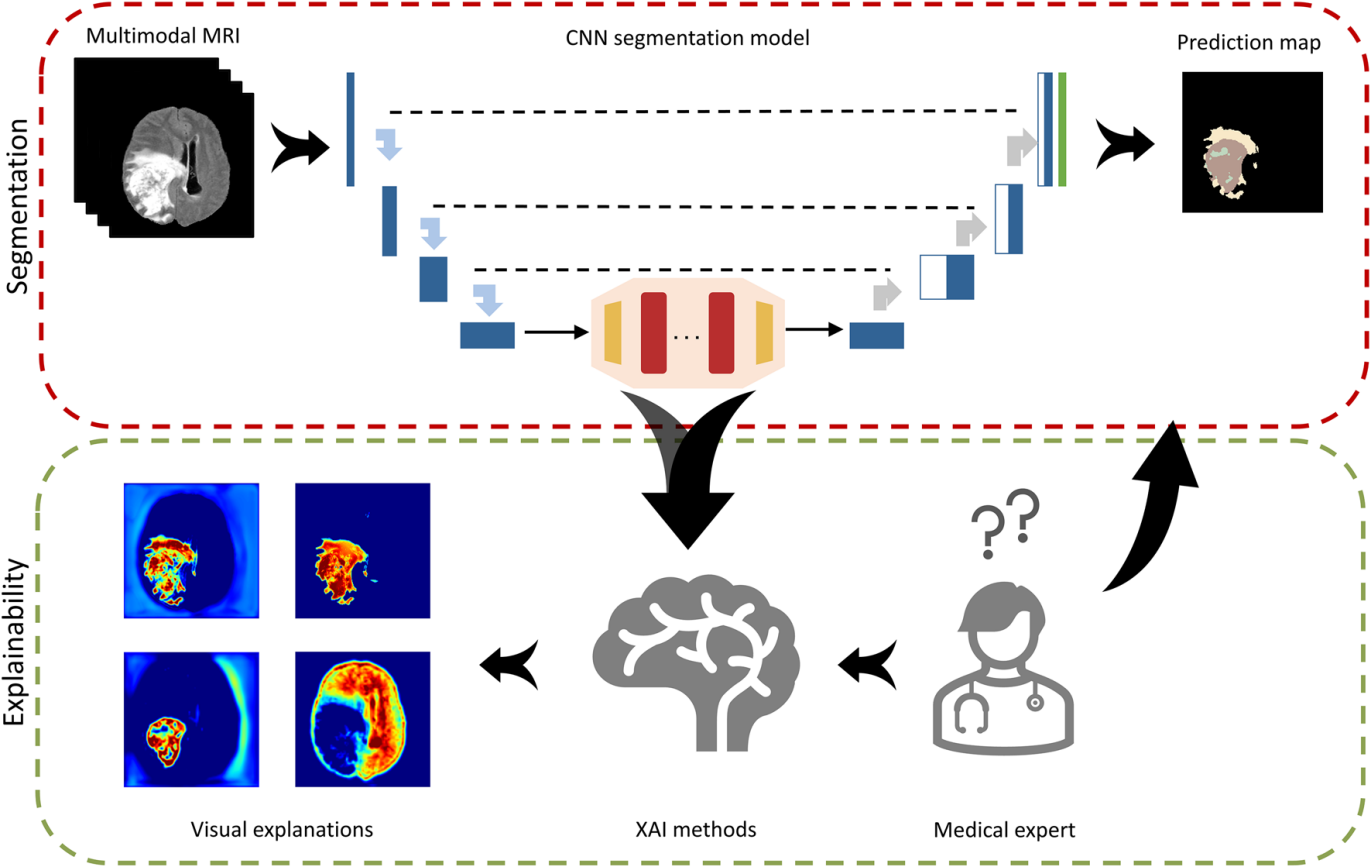
Overall proposed TransXAI pipeline for visual justification of glioma segmentation in brain MRI using a hybrid CNN-Transformer architecture.

The overall proposed gradient-based justification hybrid CNN-Transformer architecture for explainable brain lesion segmentation is depicted in Figure 4. It is a two-step approach, which combines a deep network for tumor segmentation, and an explainability generator. The first step is to segment the brain tumor boundaries from multimodal MRI data using a combined neural network with Transformer. The second step is a justification generator that is employed to provide 2D visual feature explanations. Our decision to use 2D axial slices is rooted in the pragmatic need to balance computational efficiency with clinical efficacy. Axial slices are a staple in clinical practice, providing clear views of anatomical landmarks, which is crucial for glioma boundary delineation. The following subsections describe the database used in our experiments as well as the detailed structure of the deep model and the justification generator.

### Data

**Figure 5:**
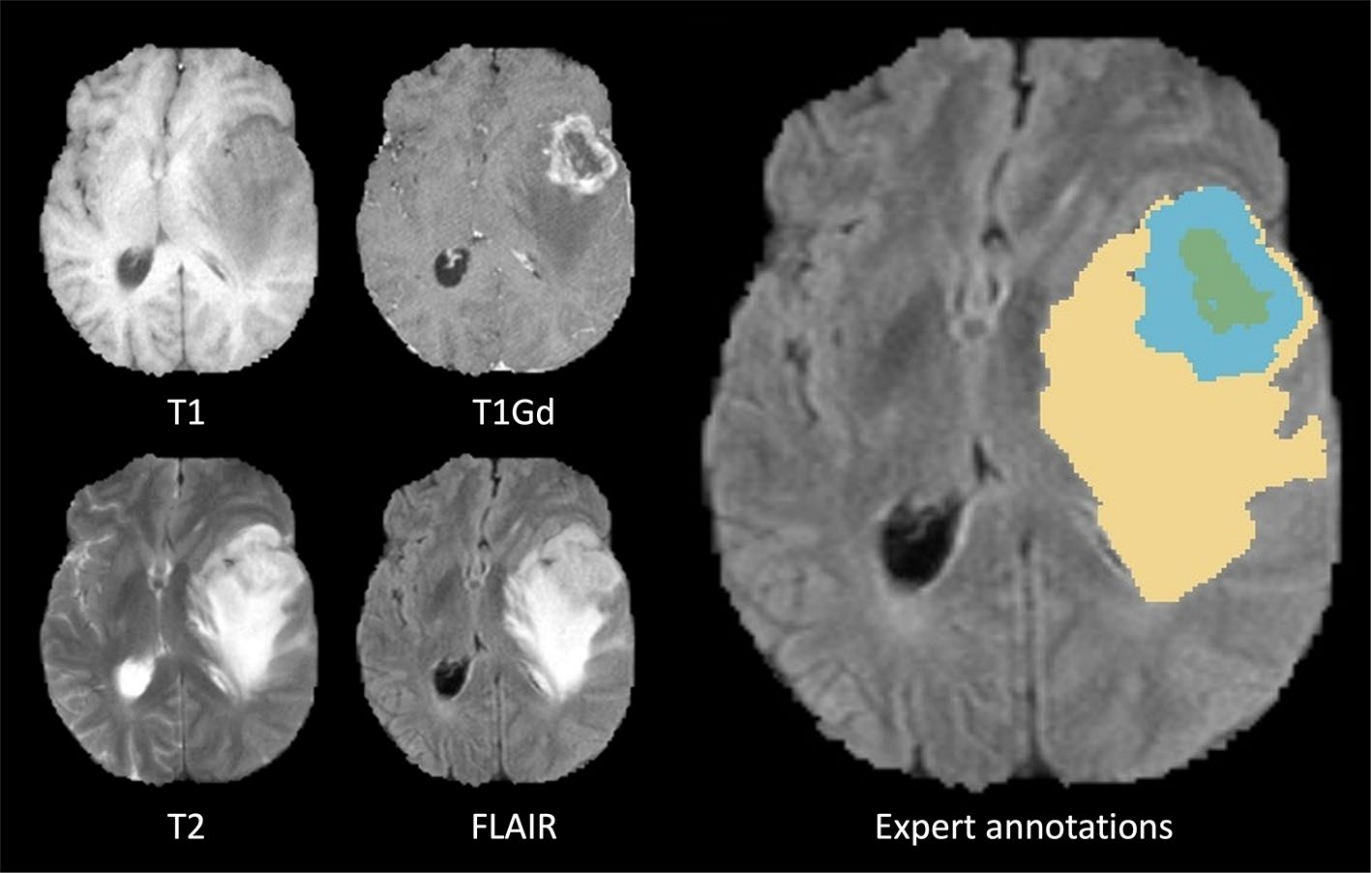
Glioma sub-regions in a sample scan from the BraTS 2019 challenge database. Image patches show the different modalities of T1, T1Gd, T2, FLAIR, and annotated expert-labeled tumor segmentation. Ground truth segmentation is provided for the enhancing tumor (*blue*) surrounding the non-enhancing necrotic tumor core (*green*) visible in T1Gd, and (b) the peritumoral Edema (*yellow*) visible in the FLAIR, respectively.

For this study, we aim to apply our explainable TransXAI approach to segment glioma in brain MRI. In our experiments, the BraTS 2019 challenge dataset^5–7^ was used including 335 training and 125 validation subjects. For every case, BraTS provides 3D pre-operative multimodal MRI scans including T1, T1Gd, T2, and FLAIR, as shown in Figure 5.

Given that the BraTS dataset was acquired from multiple institutes using various MRI scanners and following different protocols, a pre-processing stage is crucial. Therefore, to perform the tumor boundaries prediction, BraTS organizers have followed typical data pre-processing procedures^5^ including resampling to 1×1×1 mm^3^ voxel resolution, reorientation to a common coordinate system, affine registration to the same anatomical volume, and skull-stripping. Subsequently, we deploy our pre-processing pipeline as follows: first, brain pixels of each MRI volume were extracted and non-brain voxels were assigned zero. This approach leads to a closer field of view (FOV) focused on the brain, using fewer image voxels and thus reducing resource consumption. Second, z-score data normalization has been applied to the resultant volume with the standard deviation, and the center was cropped to 192 × 192 voxels.

### CNN-transformer hybrid architecture

**Figure 6:**
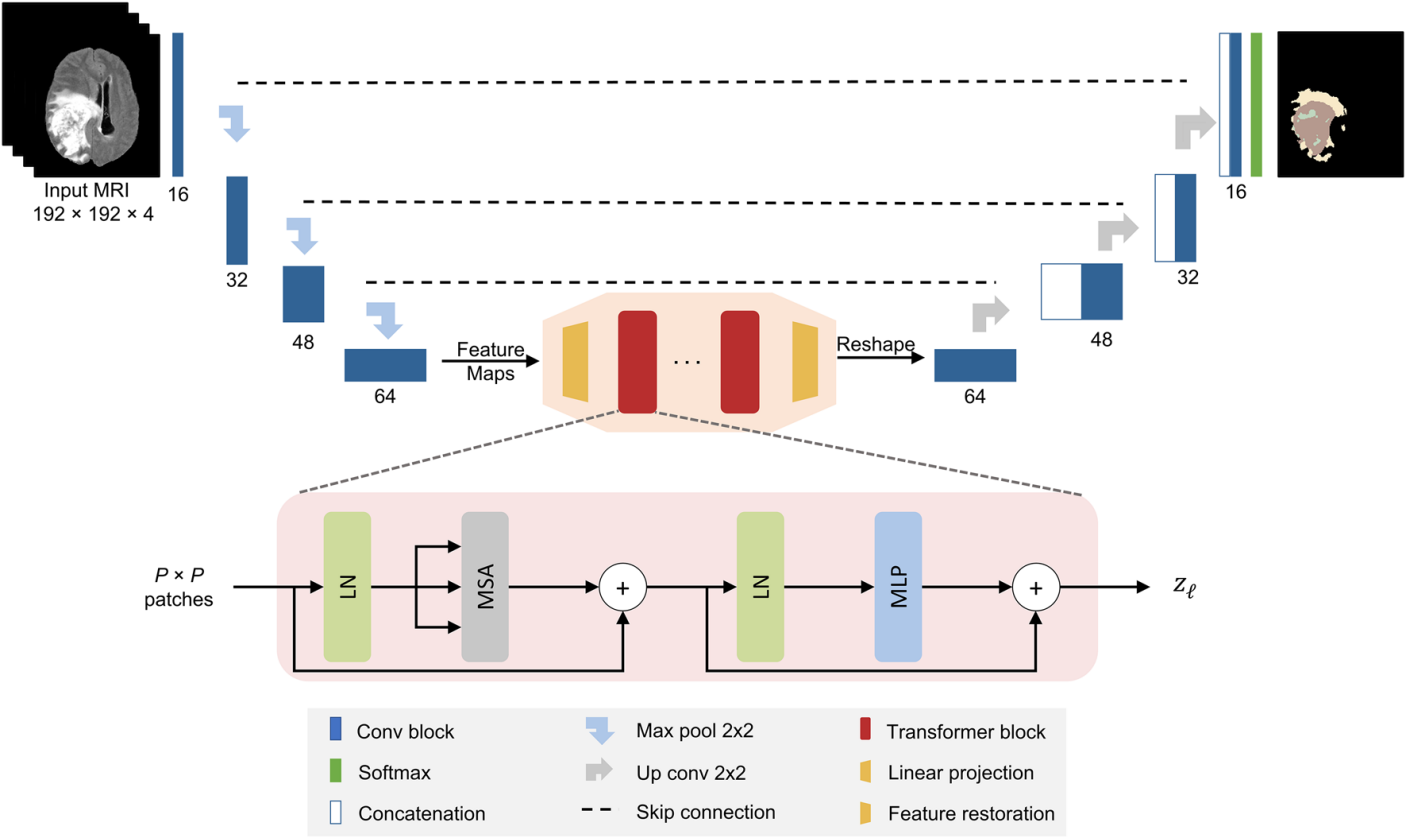
The architecture of the hybrid CNN-Transformer brain segmentation network from mpMRI volumes. The input is a 2D multimodal MRI of T1, T1Gd, T2, and FLAIR with a patch spatial resolution of 192 × 192 × 4. The network has 8 convolution neural blocks (*blue boxes*), each consisting of two successive convolutional layers 3 × 3, BN layer, and ReLU activation.

A detailed pipeline of the proposed TransXAI approach is given in Fig. 4. Given an input MRI volume $x \in {\mathbb{R}}^{H \times W \times C}$ where H × W is the spatial resolution and C number of channels (# of modalities), we first utilize modified 2D CNN, based on the widely used U-shaped encoder-decoder architecture^9,13,14^, as shown in Fig. 6, to extract high-level feature representations capturing local spatial features. The CNN-based encoder blocks first utilize 2D 3 × 3 convolutional blocks to capture the spatial and depth information. Every CNN block has a batch normalization (BN) layer between the convolution layers and ReLU activation^48,49^. For downsampling, 2 × 2 max-pooling is used to gradually extract spatial feature maps $F \in {\mathbb{R}}^{{K \times \frac{H}{8} \times \frac{W}{8} \times \frac{C}{8}}}$ (K = 32), which is 1/8 of input dimensions of H and W. Then, the Transformer encoder blocks leverage to extract the long-distance dependencies through the self-attention mechanism. The decoder is composed of 2 × 2 up convolutional layers that are applied to upscale the resultant encoded feature representation into the full-resolution segmentation maps of H × W. This hybrid CNN-Transformer strategy allows to model local context information across spatial dimensions as well as global context for volumetric segmentation.

### Transformer blocks

Figure 6 shows a number of Transformer blocks embedded in the bottleneck of our TransXAI network. Each Transformer block^21^ consists of two layers; a multi-head self-attention mechanism (MSA) and a multilayer perceptron network (MLP). A layer normalization (LN) is applied before each MSA and MLP layer in addition to employing residual connection around the output of each layer. Formally, the output $z_{\ell }$ of a layer $\ell$ can be defined as follows:

1 $$ {\mathop z\limits^{\prime }}_{\ell } = {\text{MSA}}\left( {{\text{LN}}\left( {z_{\ell - 1} } \right)} \right) + z_{\ell - 1} $$

2 $$ z_{\ell } = {\text{MLP}}\left( {{\text{LN}}\left( {z_{\ell } } \right)} \right) + {\mathop z\limits^{\prime }}_{\ell } $$

where $\ell \varepsilon \left[ {1,2, \ldots ,L} \right]$, and ${\mathop z\limits^{\prime }}_{\ell }$ is the encoded image representation.

However, using a pure Transformer as an encoder would be impractical due to its computational complexity proportional to the number of input sequences. Therefore, we follow

the ViT approach^22^ by splitting the *x* into fixed-size (*P* × *P*) patches image $x_{p} \in {\mathbb{R}}^{{P^{2} C}}$ and then reshaping each patch into a token. Note that the input to the ViT blocks is the extracted image representations by the convolutional neural encoder blocks instead of raw input images.

### Feature restoration

To match the spatial resolution of the TransXAI decoder, we introduce a feature restoration module to decode the resultant features. Specifically, the Transformer’s output sequence $z_{\ell } \in {\mathbb{R}}^{{\frac{HW}{{P^{2} }} \times C}}$ is initially reshaped to $\frac{H}{P} \times \frac{W}{P} \times C$, but the direct usage of the low-resolution Transformer encoded data (compared with the original resolution *H* × *W*) may cause loss of low-level tumor region details. To compensate for such information loss, a 1 × 1 convolutional layer is utilized which reduces the number of feature maps.

### Upsampling path

To gradually recover the abstract features and output the full-resolution segmentation map of H × W, we perform progressive upsampling using 2 × 2 up convolutional operations. Inspired by U-Net^13^, low-level encoder details are fused with high-level decoder counterparts for finer semantic information with spatial details. Finally, a multi-label softmax layer is used to estimate the final probability distribution for the output predictions.

### Explainable CNN generator

Since our main goal in this study is to investigate our hybrid CNN-based and Transformer model for brain segmentation, we integrated an efficient post-hoc XAI technique. This means that all experiments are carried out after the inference of the model, i.e., at prediction time. Principally, we applied Grad-CAM to explore the spatial attention of the network predictions over internal input features based on our trials with neurosurgeons at Ulm University Hospital.

**Figure 7:**
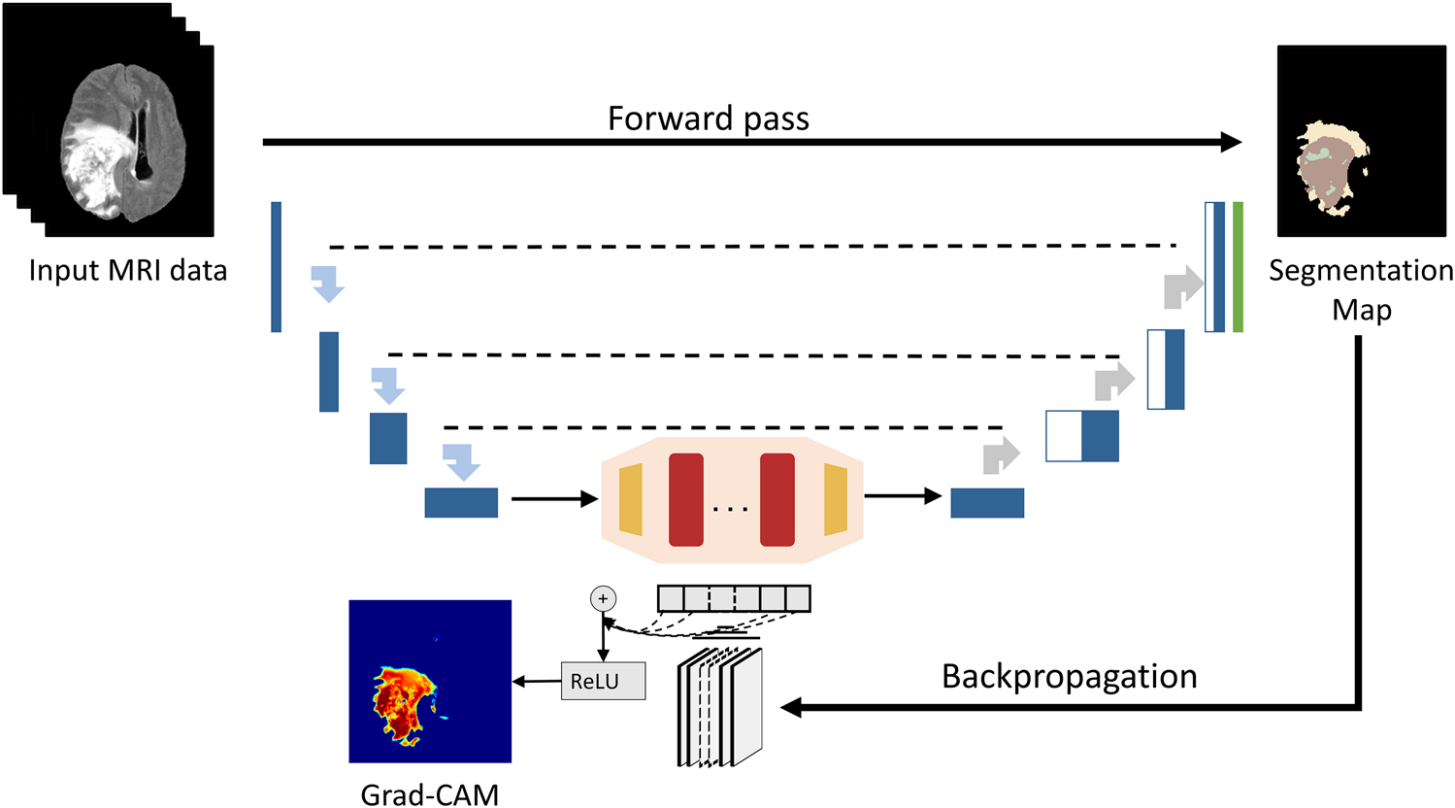
Applying grad-CAM to a sample glioma segmentation CNN model.

Grad-CAM is a generalization of the local visualization approach Class Activation Mapping (CAM)^50^ for identifying discriminative features and addressing their shortcomings. Figure 7 shows an application of Grad-CAM to segmentation neural networks, which can be applied without any architectural modifications while the model’s output layer is differentiable with respect to its input feature neurons. By using the gradient information from the last convolutional layers of the CNN, Grad-CAM can highlight the regions responsible for a particular class of interest.

Let us define the Grad-CAM heatmap as $L_{GCAM}^{c}$ which captures the important localization feature map *k* for a certain class *c* with respect to all *N* pixels (indexed by x, y). $L_{GCAM}^{c}$ is the linear combination of the forward pass activation map $A^{k}$ and the backpropagated gradient $\alpha_{k}^{c}$ with respect to the input activations followed by a ReLU activation function.

3 $$ L_{GCAM}^{c} = ReLU\left( {\mathop \sum \limits_{l} \alpha_{l}^{c} A^{l} } \right) $$

4 $$ \alpha_{l}^{c} = \frac{1}{N}\mathop \sum \limits_{x} \mathop \sum \limits_{y} \frac{{\partial y^{c} }}{{\partial A_{x,y}^{l} }} $$

### Implementation details

The 2D axial multimodal MRI images were fed into the hybrid CNN-Transformer network in randomly sampled images of 192 × 192 pixels with batch sizes of 16. For the experiments, the CNN model was implemented in TensorFlow^51^, using SGD optimizer^52^ with a momentum of 0.9, a learning rate of 8e-3, trained on a single Nvidia RTX2080Ti (11 GB) or RTX3060 (12 GB) GPU. The models were trained for 250 epochs for each fold, totaling a training time of 5 days, facilitated by a multi-GPU setup. For our experiments, we utilized the Five-fold cross-validation approach on the BraTS dataset shuffling after each epoch. The ensemble of the predictions from the five models was accomplished using the Simultaneous Truth and Performance Level Estimation (STAPLE) approach^53^, which leverages the expectation-maximization algorithm to attain comprehensive results. To alleviate the class imbalance problem in the BraTS database, we use a combination of generalized dice (GD)^54^ and categorical entropy (CE) loss functions to train the network calculated by the following equations:

5 $$ L_{Overall} = L_{GD} + L_{CE} $$

6 $$ L_{GD} = 1 - \frac{{2*\mathop \sum \nolimits_{1}^{C} W \times \mathop \sum \nolimits_{1}^{N} ys + \varepsilon }}{{\mathop \sum \nolimits_{1}^{C} W \times \left( {\mathop \sum \nolimits_{1}^{N} y + s} \right) + \varepsilon }} $$

7 $$ L_{CE} = - \frac{1}{N}\mathop \sum \limits_{1}^{N} \mathop \sum \limits_{1}^{C} y \times \log \left( s \right) $$

where *s* represents the CNN softmax predictions, *y* is the expert-labeled annotation for every tumor label, and $\in$ is a regularization parameter. The GD loss is a multi-class version of the dice loss with an adaptive weight assigned *W* to each class. Finally, in order to address the class imbalance between tumor labels and brain-healthy tissue, a range of on-the-fly spatial data augmentations have been integrated into the training process. This augmentation pipeline encompasses geometric transformations, including Horizontal-Vertical Shift (HVS), Horizontal-Vertical Flip (HSF), and randomized rotation (within the range of 0 to 20 degrees), as well as adaptive zooming (up to 20% variation). In addition, intensity augmentations, including controlled random brightness adjustments (with a maximum deviation of 20%) and judiciously introduced Gaussian noise (with a standard deviation of 0.01), further enrich the augmentation strategy. This composition of transformations is meticulously applied to the input of the multimodal MRI, enhancing the neural network's capacity to generalize effectively and ensure robust performance across diverse scenarios. For explainability experiments, we utilized the Grad-CAM implementation from NeuroXAI framework^55^.

### Experimental design and procedure

In encoder-decoder networks, like TransXAI, generated saliency maps for one of the last encoder layers are smooth and do not capture feasible information in our segmentation problem. This is because these layers generate the smallest feature dimensions in the network and intensive upscaling is required to match the output prediction map. In contrast, selecting one of the last layers (e. g. the output layer) from the decoder network provides a higher-resolution feature map showing detailed features of the segmentation process since these layers are combined with the encoder layers through concatenation. Moreover, by incorporating the output layer into the explanation generation process, we solve the limitation of Grad-CAM for generating low-resolution heatmaps.

Furthermore, our applied XAI generator is post-hoc in the sense that it provides explanations after obtaining the model predictions, instead of being inserted into the network architecture itself. Therefore, all our explainability experiments have been done after the training of the segmentation network. Pre-trained weights for the segmentation were used for generating the heatmaps of the used XAI methods, i.e., Grad-CAM.

Since segmentation networks pinpoint the localized region of brain tumor regions, providing visual saliency maps of the output layer alone does not help in making the network transparent. Therefore, to better investigate the behavior of the deep model and to determine how spatial information flows inside the internal layers, we conducted four main experiments to extend the explainability approach as follows:

- Quantitative evaluation on the BraTS validation database and comparison with SOTA 2D and 3D methods.
- Identifying the contribution of each MRI input modality in the final predicted tumor sub-components.
- Interpreting the CNN layers using XAI to reveal how the network represents information in the internal filters.
- Clinical feedback on the proposed method from our clinical collaborators.TransUNetTiny2_wcross_loss
- Detection of failure nodes of the TransXAI model and analysis of the reasons behind that.

## Conclusion

This article demonstrated our successful TransXAI as a 2D generic explainability generator for interpreting the performance of multimodal CNN for brain glioma segmentation using MRI scans. Our proposed TransXAI holds a competitive position among other SOTA methods by achieving mean dice scores of 0.88, 0.78, and 0.75 on the WT, TC, and ET sub-regions. This balanced performance highlights its potential in clinical applications alongside other advanced methods. However, visual pixel-based representations are not enough to give meaningful interpretable information, and therefore, we conducted extensive experiments to provide interpretability by evaluating their clinical significance. The obtained results supported our technical research work to realize that deep neural models behave in a human-understandable manner and are consistent with the surgical experts’ domain knowledge. The decision-making clarity provided by TransXAI's explainability promotes trust among clinicians, ensuring that the model's predictions are not only accurate but also understandable and aligned with clinical expertise.

For future work, the generalization architecture of our proposed TransXAI can be extended by adding new CNN models. Our future research will study the integration of a 3D architecture, with the aim of investigating its potential to further enhance performance and accuracy. The consideration of 3D volumetric data could potentially capture spatial relationships and contextual information that are inherently present in medical images, potentially leading to improved segmentation outcomes. Further studies should explore utilizing concept activation maps and feeding them back to the neural network as on-demand deep supervision. That will provide additional guidance to the network and thus enhance the overall accuracy of assisting the surgeons during interventional procedures.

## Data Availability

BRATS 2019 dataset analyzed during the current study is included in this article https://doi.org/10.48550/arXiv.1811.02629 and is available through the Image Processing Portal of the CBICA@UPenn (IPPipp.cbica.upenn.edu). This platform features downloading of the dataset, as well as the automatic evaluation of the submitted results.

## Code availability

The source code and the pre-trained models for this study will be publicly available upon acceptance on GitHub at the following repository: https://github.com/razeineldin/TransXAI.
